# Physiology and Transcriptome Analysis of Exogenous GA_3_ Effects on the Seed Germination of *Phyllostachys edulis*

**DOI:** 10.3390/cimb47090686

**Published:** 2025-08-25

**Authors:** He Liao, Shinan Liu, Yuansong Li, Qiancheng Yang, Ying Hu

**Affiliations:** Guangxi Colleges and Universities Key Laboratory for Cultivation and Utilization of Subtropical Forest Plantation, College of Forestry, Guangxi University, Nanning 530004, China; 1936200134@st.gxu.edu.cn (H.L.); 2309392019@st.gxu.edu.cn (Y.L.); 20240173@gxu.edu.cn (Q.Y.); huying19863008@163.com (Y.H.)

**Keywords:** *Phyllostachys edulis*, seed germination, exogenous GA_3_, transcriptome, physiological indexes

## Abstract

*Phyllostachys edulis* is a vital bamboo resource in China, known for its economic benefits and ecological functions. However, under natural conditions, its seed germination rate is very low. Exogenous gibberellin (GA) directly supplements endogenous GA levels, while paclobutrazol (PAC) is an inhibitor of GA biosynthesis that can prevent seed germination. Preliminary experiment indicated that a treatment of 50 mg/L GA_3_ markedly enhanced the germination rate of *P. edulis* seeds, whereas 50 μmol/L PAC had an opposite function. To study the exogenous GA_3_ effects on the seed germination of *P. edulis*, seeds were soaked in ddH_2_O (CK), Gibberellic acid 3 (GA_3_), and PAC solutions for 24 h, respectively. Then, we analyzed and compared the physiology, biochemistry, and transcriptome at different germination stages. The results demonstrated that exogenous GA_3_ treatment significantly reduced the contents of starch and soluble protein while increasing the levels of soluble sugar by inducing the activities of β-amylase and protease, respectively. In addition, the activities of superoxide dismutase (SOD), polyphenol (PPO), and ascorbate peroxidase (APX) were enhanced to eliminate ROS during seed germination under exogenous GA_3_ treatment compared to CK and PAC treatments. Moreover, the endogenous levels of GA_3_ and JA were found to be higher in exogenous GA_3_-treated seeds than those in CK and PAC-treated seeds. Furthermore, RNA-seq results revealed that the expressions of 10 related genes are consistent with the observed physiological changes. In summary, exogenous GA_3_ effectively accelerated the seed germination of *P. edulis* by influencing storage reserves, antioxidant enzymes activity, and endogenous hormone through the coordinated transcriptional regulation of related genes. These findings provide novel insights into the regulation mechanisms of exogenous GA_3_ on the seed germination of *P. edulis*.

## 1. Introduction

Seed germination represents a pivotal juncture in the plant life cycle, indicating the shift from a dormant state to a metabolically dynamic phase of growth and development [1,2]. This intricate process encompasses a range of fundamental metabolic activities, including nutrient utilization, water absorption, and respiratory metabolism. During germination, stored macromolecules within the seed including lipids, proteins, and starch are hydrolyzed to provide the essential energy required for this transition [3]. Simultaneously, relevant enzymes are activated, and plant hormones undergo changes in concentration through accumulation or degradation [4,5]. Seed germination is impacted by a combination of external environmental conditions and internal physiological factors. Among them, plant hormones exert an essential role in precisely regulating the germination process by integrating and responding to these diverse signals.

Gibberellic acid (GA) is a crucial hormone in plants, known for its role in promoting seed germination by enhancing the release from dormancy, as demonstrated in various plant species [6,7]. In the early stages of seed germination, GA can mobilize the stored substances in seeds and stimulate the embryo expansion [8]. Furthermore, it facilitates the physiological transition from embryo to plant development, potentially mediated by a chromatin remodeling factor known as PICKLE (PKL) [9]. In the later stages of seed germination, GA can enhance the expression of hydrolases that soften the barrier tissues, thereby reducing the mechanical resistance at the tip of the radicle and facilitating radicle emergence through the seed coat [9,10]. Thus, exogenous GA_3_ is widely applied to induce seed germination. Numerous studies indicate that exogenous GA_3_ can stimulate the synthesis of amylase and protease in seeds, promoting the conversion of starch, protein, and fat into soluble protein and sugar, consequently facilitating seed germination [11,12]. In addition, exogenous GA_3_ increases the levels of promoters including Gibberellic acid 3 (GA_3_), jasmonate (JA), and indole acetic acid (IAA) while also promoting the degradation of ABA in plant seeds, thus regulating the synergistic and antagonistic interactions among these endogenous hormones [13,14].

The promotion of seed germination by GA is primarily caused by GA metabolism and GA signal transduction [6,15,16]. Recent advancements in plant functional genomics have significantly enhanced our understanding of GA in grasses and *Arabidopsis thaliana*. The key enzyme genes involved in GA biosynthesis and metabolism have been thoroughly elucidated, and crucial components of the GA signaling transduction pathway have been successfully isolated [15,17]. The biosynthetic enzyme genes *CPS*, *KS*, *KO*, *GA20ox*, *GA3ox*, along with the metabolic enzyme gene GA2ox are the key genes in the GA metabolic pathway determining seed germination [18,19]. In addition, some key genes in the GA signaling pathway also impact seed germination [20,21,22]. DELLA proteins serve as critical negative regulators in the GA signaling pathway. Homologs of DELLA have been cloned in various plants, such as SLN1 in barley, SLR1 in rice, and GAI, RGA, RGL1, RGL2, and RGL3 in *Arabidopsis thaliana* [23,24], exhibiting a high degree of conservation across different plants. The GA signal operates through its receptor GID1, which first binds to active GAs and subsequently interacts with the DELLA protein SLR1. The interaction causes the degradation of SLR1, triggering a series of downstream reactions [25,26]. The loss of *GID1* function in rice and *gid1* triple mutant in *A. thaliana* are both unresponsive to the promoting effect of GA [20,25,26,27], while overexpression of *GID1* leads to a GA hypersensitive phenotype. Therefore, the GA signal promotes seed germination by effectively regulating seed metabolic activities and the expression of related genes.

Over the past three decades, both the total area and carbon storage capacity of China’s bamboo forests have expanded steadily [28]. Among them, *Phyllostachys edulis* stands out as the most ecologically and economically important species, occupying ~70% of the nation’s bamboo forest area and generating an annual economic value of approximately 5 billion USD [28,29]. *P. edulis* spreads predominantly via clonal transplanting, allowing rapid colonization of neighboring areas; yet this vegetative strategy fosters stand structural uniformity and erodes understory diversity [30]. In recent years, the flowering phenomenon of *P. edulis* has been observed annually in northern Guangxi, China. Mass flowering can result in the degradation and even the death of bamboo forests [31]. Seed-based propagation is therefore indispensable for reconstructing and rejuvenating *P. edulis* forests [30]. Nevertheless, the seeds of *P. edulis* are known for their low germination rates and short lifespan. Previous studies demonstrated that the carbohydrate degradation is a primary energy source for seed germination of *P. edulis* [32]. Moreover, exogenous GA_3_ can significantly elevate the germination rates by accelerating the decomposition of starch and fat [33]. Different from rice and *A. thaliana*, homologous *GID1*- and *DELLA*-related genes are not expressed, but the expression of *PIF4*, *PIF5*, *GA3ox2*, *GA2oxs*, etc., were up-regulated in *P. edulis* after GA_3_ treatment [33]. This finding suggests that the mechanism by which exogenous GA_3_ functions on the seed germination of *P. edulis* may be different. Nonetheless, this mechanism still needs to be further explored. Therefore, this study aims to investigate the effects of exogenous GA_3_ on *P. edulis* seeds at morphological, physiological, and transcriptomic levels by comparing the double-distilled water (ddH_2_O), GA_3_, and paclobutrazol (PAC) treatments. Furthermore, this research is essential for uncovering the key mechanisms involved in the response of *P. edulis* seeds to exogenous GA_3_ and provides a theoretical basis for the enhancement of breeding programs.

## 2. Materials and Methods

### 2.1. P. edulis Characteristics

*P. edulis* belongs to the genus *Phyllostachys* from the Poaceae family, which is the most ecologically, economically, and culturally significant wood bamboo of subtropical China. It has a unique rhizome-dependent proliferation system that runs radially from the mother plant. In addition, *P. edulis* only flowers once in its lifetime and dies after seed production.

### 2.2. Plant Material and Treatment

**Figure 1:**
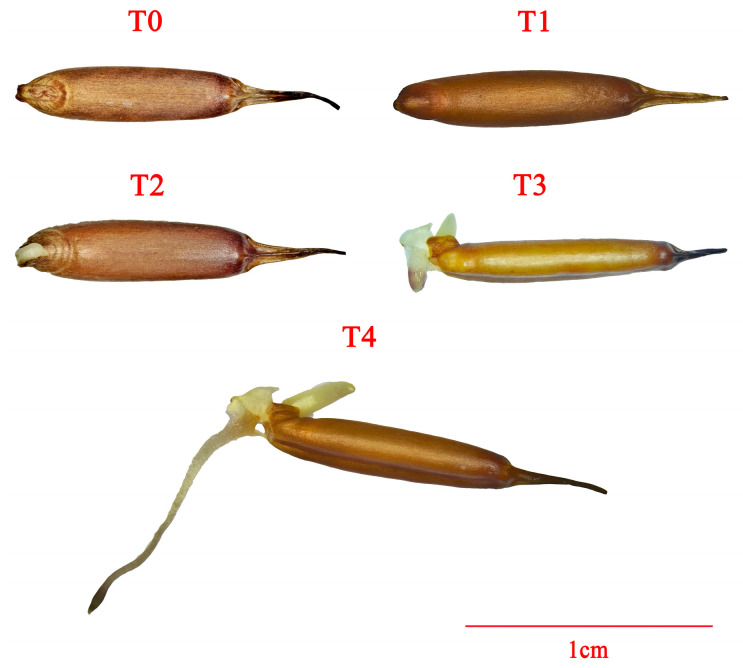
Morphology of *P. edulis* seed at different stages during the germination.

In August 2023, the caryopses of *P. edulis* were collected from Lingchuan County, Guilin City, Guangxi Zhuang Autonomous Region. The caryopses without glume (named seeds) were used for further experiments. To guarantee uniformity and viability, only plump seeds were selected. These seeds were surface-sterilized by immersing in a 75% ethanol solution for 5 min, followed by five rinses with sterile distilled water. After surface sterilization, the seeds were soaked separately in double-distilled water (ddH_2_O, CK), 50 mg/L GA_3_ solution, and 50 μmol/L paclobutrazol (PAC, an inhibitor of gibberellin biosynthesis) solutions for 24 h, with a weight range of 1.48–1.49 g. Following the soaking process, the seeds were evenly distributed on double-layer filter paper in Petri dishes and then placed in an incubator at 25 °C with 65–70% relative humidity under dark conditions for germination. Each Petri dish contained 50 seeds, and germination data were recorded with three biological replicates. Seed samples were collected at stages 0 h (T0, dry seed), 24 h (T1, soaking), 48 h (T2, whitening), 72 h (T3, radicle emergence), and 96 h (T4, when radicle length equals seed length) from CK, GA_3_, and PAC treatments to serve as experimental materials (Figure 1). Regarding each treatment, three biological replicates were prepared. The collected samples were immediately immersed in liquid nitrogen to rapidly freeze them and then stored at −80 °C for further analysis.

### 2.3. Measurements of Germination Parameters

Seeds were considered to be germinated when the radicle broke through the seed coat and reached a length equal to seed length. The germination process was deemed complete when the daily germination rate remained below 1% for three consecutive days. Then the germination parameters were calculated using the following equations [34].

(1) $$Germinationpercentage\;(GP)=\frac{Number\;of\;germinated\;seeds}{Number\;of\;total\;tested\;seeds}\times 100\%$$

(2) $$Germinationenergy\;(GE)=\frac{Number\;of\;germinated\;seeds\;on\;the\;day\;of\;peak\;germination}{Number\;of\;total\;tested\;seeds}\times 100$$

(3) $$Germinationindex\;(GI)=\Sigma \frac{Gt}{Dt}$$

(4) $$Meangerminationtime\;(MGT)=\frac{Germination\;time\;for\;all\;seeds\;}{Number\;of\;total\;germinated\;seeds}$$

Gt is the number of germinated seeds on day t and Dt is the day t.

### 2.4. Determination of Physiological and Biochemical Parameters

Soluble sugar and starch contents were measured using the anthrone colorimetry [35], while soluble protein content was determined using the coomassie brilliant blue staining [36]. The acid phosphatase activity was determined through the p-nitrophenyl phosphate (pNPP) method [37], while the protease activity was measured using the Folin–phenol method [38]. In addition, the α-amylase and β-amylase activity were determined following the instructions of the assay kits from Suzhou Grace Biotechnology Co., Ltd. (Suzhou, China).

Superoxide dismutase (SOD) activity was measured by nitroblue tetrazolium (NBT) method [39]. Polyphenol oxidase (PPO) activity was measured using the catechol method [40]. Ascorbate peroxidase (APX) activity was measured by decreasing ascorbate at 290 nm [41]. Malondialdehyde (MDA) content was quantified using the thiobarbituric acid (TBA) method [42]. Endogenous hormone ABA, GA_3_, IAA, and JA contents in the seeds of *P. edulis* at different stages were examined by LC-MS/MS internal standard method [43].

### 2.5. RNA Isolation and Transcriptome Profiling

As described in Section 2.1, seed samples from each treatment were collected at various germination stages. Total RNA was extracted using the EASYspin Plus Plant RNA Kit (Aidlab Biotechnologies Co., Ltd., Beijing, China). The quality of the extracted RNA was assessed with a ScanDrop 100 (Analytik Jena, Thuringia, Germany) and 1.2% agarose gel electrophoresis. Libraries were constructed using the NEBNext Ultra II RNA Library Prep Kit for Illumina (New England BioLabs, Ipswich, MA, USA) and subsequently sequenced on the Illumina NovaSeq 6000 platform (Illumina, San Diego, CA, USA). Raw FASTQ data were filtered to obtain clean reads, which were subsequently aligned to the *P. edulis* reference genome [44] using HISAT2 v2.0.5 software. Gene expression levels were quantified as Fragments Per Kilobase of transcript per Million fragments mapped (FPKM).

Differential gene expression analyses among *P. edulis* seeds subjected to control (CK), GA_3_, and PAC treatments at different germination stages were carried out using DESeq (v1.20.0) software [45]. Pairwise comparisons were made between CK, GA_3_, or PAC treatments at the same time points. Genes with significant expression changes were identified as differentially expressed genes (DEGs) if they satisfied the threshold criteria of |log_2_FoldChange| > 1 and *p*-value < 0.05. Gene Ontology (GO) enrichment analysis was performed using GOseq software (http://www.geneontology.org/). KEGG pathway enrichment analysis was performed using KOBAS software (http://www.kegg.jp/) to identify significantly enriched metabolic and signaling pathways. GO terms and KEGG pathways with q-value ≤ 0.05 were considered significantly enriched. Heatmaps were finished on the BioDeep Platform (https://www.biodeep.cn).

### 2.6. Statistical Analysis

An analysis of variance (ANOVA) of one-way followed by Duncan’s multiple range test were performed with IBM SPSS Statistics 26.0 (IBM, New York, NY, USA), and data were presented as the mean ± standard deviation (SD) from three independent biological replicates. Principal component analysis (PCA) was conducted with Origin 2025 (OriginLab, Northampton, MA, USA). All figures were generated using Origin 2025.

## 3. Results

### 3.1. Germination of P. edulis Seeds Was Stimulated by Exogenous GA3

As shown in Table 1, the application of GA_3_ significantly elevated the GP of seeds to 52%, which surpassed the CK, exhibiting a GP of 41%. Although the GE revealed no significant difference between the GA_3_ treatment (38.89%) and CK treatment (36.60%), the GI was significantly higher in the GA_3_-treated seeds compared with the CK group. Furthermore, although not statistically significant, the MGT of seeds treated with GA_3_ was slightly shorter than that of the CK group. In contrast, seeds treated with PAC exhibited a complete loss of germination ability, with all germination parameters being recorded as zero. These findings collectively suggested that exogenous GA_3_ effectively enhances GP and GI, therefore significantly improving the overall germination capacity of the *P. edulis* seeds.

### 3.2. Effects of Exogenous GA_3_ on Storage Substances at Different Germination Stages of the Seeds

**Figure 2:**
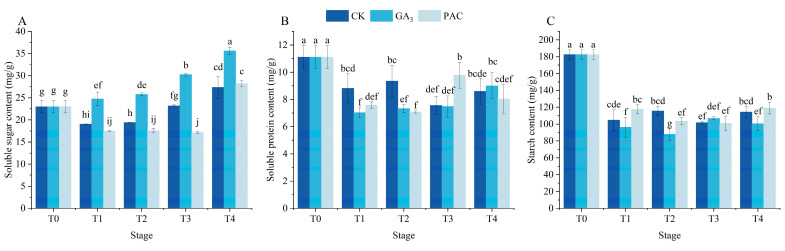
Changes in storage material including soluble sugar content (**A**), soluble protein content (**B**), and starch (**C**) during seed germination of *P. edulis* under different treatment. Vertical bars represent means value ± SE (*n* = 3). Data in the same column followed by different letters indicate significant differences among the treatments at the *p* < 0.05 level.

The soluble sugar content in GA_3_ treatment increased over time and was significantly higher than that in CK and PAC treatments (*p* < 0.05) (Figure 2A). The soluble protein content in all treatments decreased after the T1 stage, with the greatest decrease being observed in GA_3_ treatment (Figure 2B). Subsequently, the soluble protein content gradually increased, surpassing that of CK treatment at the T4 stage. The seeds of *P. edulis* are classified as starch-storing seeds, where the degradation of starch exerts a vital role in seed vigor and germination. The results demonstrated that starch content decreased after the T1 stage in all treatments (Figure 2C). In the GA_3_ treatment, starch content was significantly lower at the T1, T2, and T4 stages relative to the CK and PAC treatments (Figure 2C).

### 3.3. Effects of Exogenous GA_3_ on Hydrolase During the Germination Process

**Figure 3:**
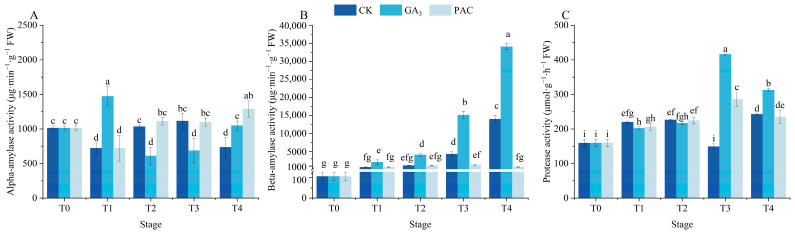
Changes in α/β-amylases (**A**,**B**) and protease activity (**C**) during seed germination of *P. edulis* under different treatment. Vertical bars represent means value ± SE (*n* = 3). Data in the same column followed by different letters indicate significant differences between different treatment at the *p* < 0.05 level.

The activities of protease and α/β-amylases were measured to assess the impacts of exogenous GA_3_ on the germination of *P. edulis* seeds (Figure 3). The α-amylase activity significantly increased after soaking in GA_3_ for 24 h, followed by a gradual decrease and then increased during the germination stages (Figure 3A). Differently, the α-amylase activity significantly decreased after soaking in CK and PAC for 24 h, which increased and subsequently decreased under the CK treatment, reaching its peak at the T3 stage (Figure 3A). Throughout the germination process, β-amylase activity exhibited a gradual increase under GA_3_ and CK treatments, while maintaining a stable value under PAC treatment (Figure 3B). In general, β-amylase activity under GA_3_ treatment was far more than under other treatments. In addition, protease activity initially increased and then decreased under both GA_3_ and PAC treatments, reaching the peak at the T3 stage (Figure 3C). However, under CK treatment, protease activity significantly decreased, reaching the nadir at the T3 stage before rising to the peak at the T4 stage. Obviously, protease activity under GA_3_ treatment was significantly higher than the other two treatments at the T3 and T4 stages.

### 3.4. Effects of Exogenous GA_3_ on the Contents of Antioxidant Enzymes and MDA During the Germination Process

**Figure 4:**
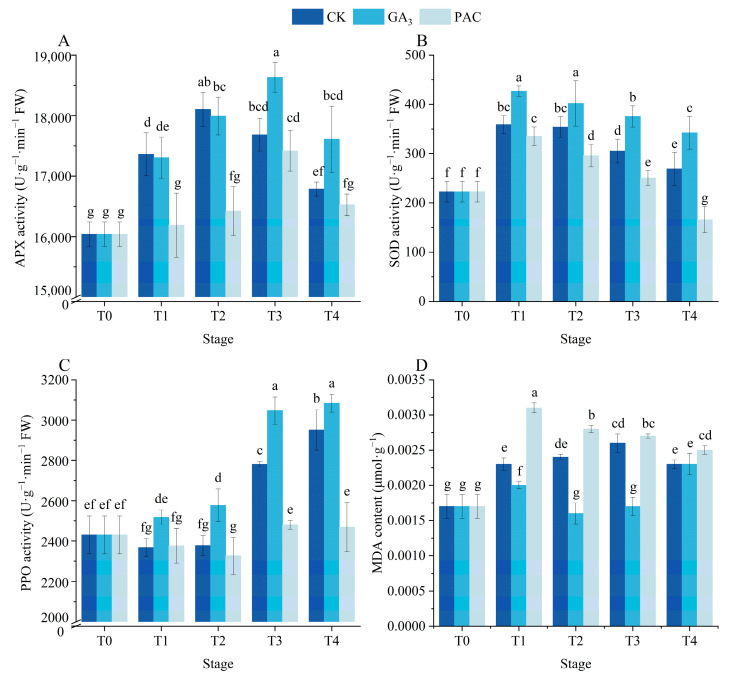
Changes in the activities of APX (**A**), SOD (**B**), PPO (**C**), and content of MDA (**D**) during seed germination of *P. edulis* under different treatment. Vertical bars represent means value ± SE (*n* = 3). Data in the same column followed by different letters indicate significant differences between different treatment at the *p* < 0.05 level.

The activities of SOD, PPO, and APX, along with the MDA content were determined to evaluate the impact of GA_3_ treatment on the antioxidant enzyme system during seed germination (Figure 4). APX activity exhibited a notable increase under GA_3_ and CK treatments (*p* < 0.05), initially rising before declining (Figure 4A). During the T1 and T2 stages, APX activity under CK treatment was higher than under GA_3_ and PAC treatments (Figure 4A). However, at the T3 and T4 stages, the APX activity under GA_3_ treatment surpassed that of CK and PAC treatments, with no significant difference being observed between CK and PAC treatments (Figure 4A). Following soaking treatments, SOD activity significantly increased across all groups and subsequently gradually declined over time (*p* < 0.05, Figure 4B). Meanwhile, PPO activity exhibited a steady increase over time in GA_3_ treatments (Figure 4C). The MDA content significantly increased after soaking, subsequently exhibiting distinct patterns at the germination stages among the three treatments (*p* < 0.05, Figure 4D). Afterwards, the MDA content under GA_3_ treatment initially decreased and later increased, while an opposite trend was observed under CK treatment (Figure 4D). In contrast, MDA content in PAC-treated seeds gradually decreased. Overall, during most stages of germination, SOD and PPO activities across different treatments followed the order as GA_3_ > CK > PAC, while the MDA content showed the opposite order as PAC > CK > GA_3_ (Figure 4B,C,D).

### 3.5. Effects of Exogenous GA_3_ on Endogenous Hormones During the Germination Process

**Figure 5:**
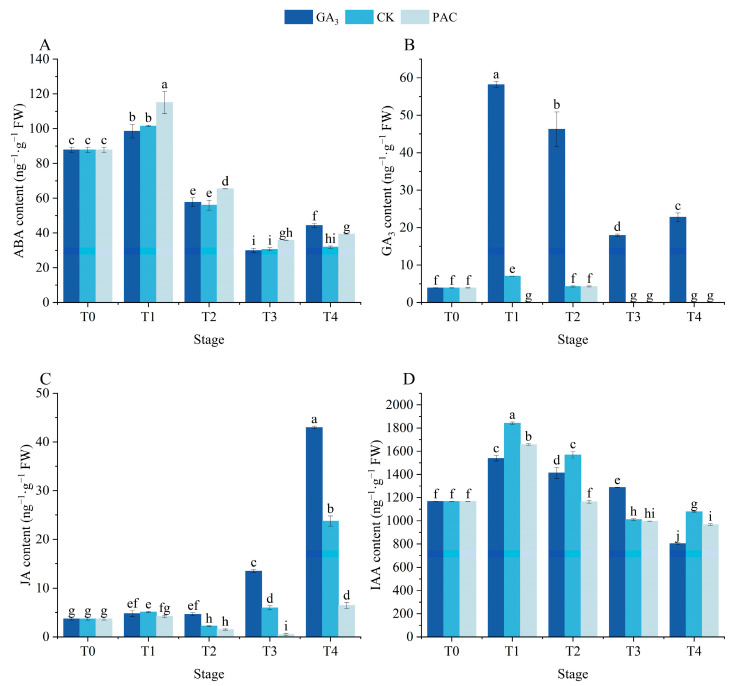
Changes in the contents of ABA (**A**), GA_3_ (**B**), JA (**C**), and IAA (**D**) during the seed germination of *P. edulis* under different treatment. Vertical bars represent means value ± SE (*n* = 3). Data in the same column followed by different letters indicate significant differences between different treatment at the *p* < 0.05 level.

The contents of ABA, GA_3_, IAA, and JA were explored to evaluate the impact of GA_3_ treatment on the endogenous hormones during the germination process (Figure 5). The ABA content exhibited similar trends across the three treatments, with a significant increase after soaking (*p* < 0.05, Figure 5A). Next, it gradually decreased and then increased during the germination stages. No significant differences in ABA content were observed between GA_3_ and CK treatments from the T1 to T3 stages, while the PAC treatment exhibited significantly higher levels than other two treatments. However, the increase at the T4 stage was shown to be higher in the GA_3_ treatment. The endogenous GA_3_ content significantly increased after soaking under both GA_3_ and CK treatments, while it decreased under PAC treatment (Figure 5B). Afterwards, the GA_3_ treatment first decreased and then increased, remaining much higher than both CK and PAC treatments. From the T2 to T4 stages, there existed no difference between CK and PAC treatments, both of which dropped to zero after the T3 stage. The JA content significantly increased in both GA_3_ and CK treatments after soaking (*p* < 0.05, Figure 5C). Subsequently, the GA_3_ treatment revealed no significant differences between T1 and T2 stages, while it increased sharply and reached its peak at the T4 stage, significantly higher than that of CK and PAC treatments. From T1 to T4 stages, both CK and PAC treatments initially decreased and then increased, reaching the nadir at the T2 and T3 stages, respectively. The IAA content exhibited similar trends across three treatments, first increasing and then decreasing. At the T1, T2, and T4 stages, the IAA content in the GA_3_ treatment was lower than that in the CK treatment (Figure 5D). These results suggested that exogenous GA_3_ induced seed germination by modulating the endogenous GA_3_ and JA levels.

### 3.6. Principal Component Analysis

**Figure 6:**
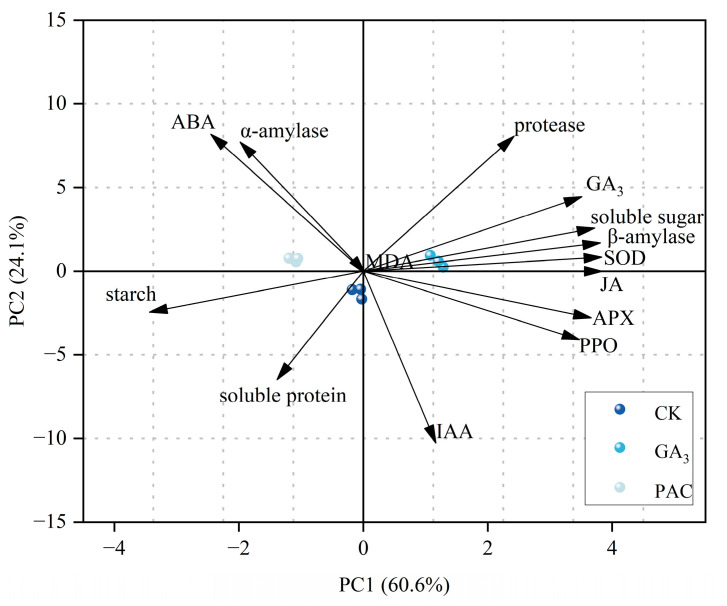
Biplot of the principal components analysis for 14 physiological parameters under different treatments. Arrows represent PCA loadings, with their direction indicating the correlation of each parameter with the principal components, and their length denoting the magnitude of the contribution.

PCA was conducted based on various physiological and biochemical parameters to evaluate the effects of different treatments. The results showed that the first two principal components accounted for 84.7% of the variance, with PC1 explaining 60.6% and PC2 explaining 24.1%, respectively (Figure 6). The CK, GA_3_, and PAC treatments were clearly separated along PC1 axis, with GA_3_ treatment positively correlated with PC1 and PAC treatment negatively correlated. Most enzyme activities, endogenous hormones, and storage substances contributed strongly to PC1, with SOD, APX, PPO, and β-amylase, as well as JA, GA_3_, and soluble sugar exhibiting positive loadings, while starch showed negative loadings (Table A1). This suggests that PC1 can effectively reflect germination capacity, which includes antioxidant capacity, endogenous hormone level, and the efficiency of storage substance utilization. By contrast, in PC2, parameters such as SOD, β-amylase, JA, soluble sugar, and starch had relatively low contributions, providing limited differentiation among these treatments. Overall, differences in germination capacity among treatment groups were primarily distinguished by PC1, with exogenous GA_3_ enhancing seed germination and PAC exhibited an inhibitory effect.

### 3.7. RNA Sequencing and Gene Annotation of P. edulis Seed Transcriptome

**Figure A1:**
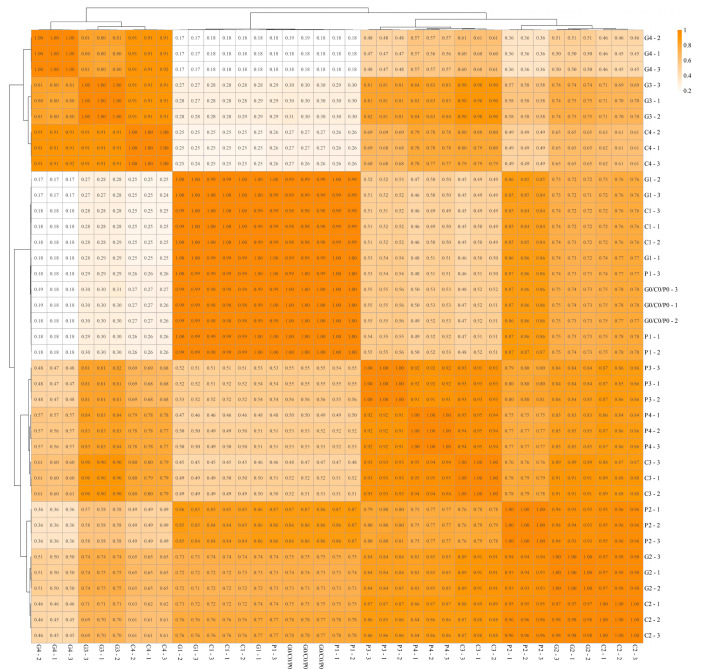
Correlation test of gene expression levels among different samples.

To comprehensively understand the effects of exogenous GA_3_ on seed germination, samples from various soaking conditions including GA_3_, CK, and PAC were selected at each sampling point (0 h, 24 h, 48 h, 72 h, and 96 h) for transcriptome assays. In total, 39 cDNA libraries were constructed from the whole seeds of *P. edulis* to investigate the gene expression profile at the transcriptome level. A total of 251.50 Gb clean reads were generated, with a Q30 value of approximately 94% for each sample (Table A2). The Pearson Correlation Coefficient (R) was employed to evaluate the consistency among the three biological replicates, with all R^2^ values exceeding 0.99 (Figure A1), demonstrating a robust correlation between the replicates of each sample.

### 3.8. Comparative Analysis of DEGs in P. edulis Seeds with Exogenous GA3 Treatment

**Figure 7:**
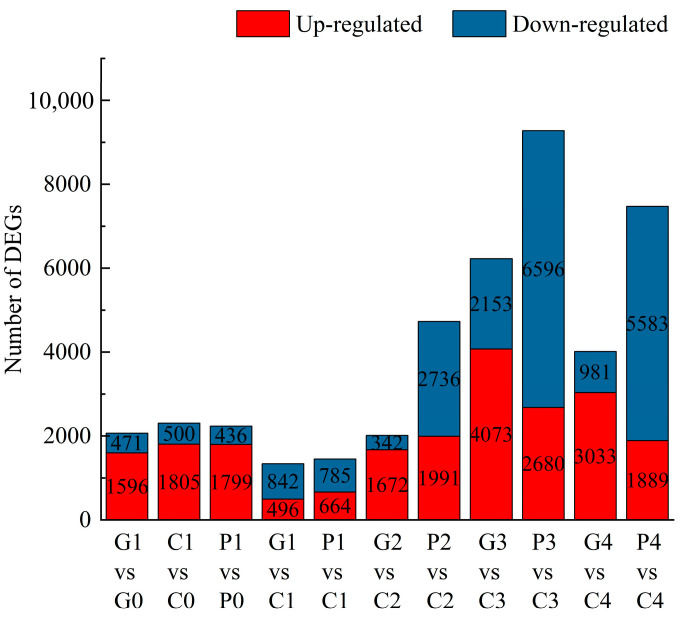
Statistics on the number of upregulated and downregulated DEGs in different comparison groups. G, C, and P represent GA_3_, CK, and PAC treatments, while 1–4 represent T1–T4 stages, respectively. Red indicates the number of up-regulated DEGs, while Blue indicates the number of down-regulated DEGs.

**Figure 8:**
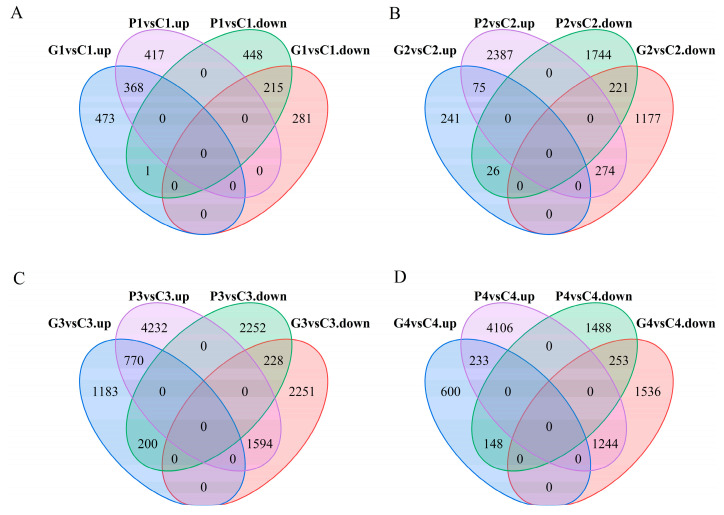
The number of up-regulated and down-regulated DEGs in different comparison groups at the stages of T1, T2, T3, and T4. (**A**) Venn diagram showing the overlapping up-regulated DEGs of GA_3_ vs. CK (G1 vs. C1) and PAC vs. CK (P1 vs. C1), and down-regulated DEGs of GA_3_ vs. CK (G1 vs. C1) and PAC vs. CK (P1 vs. C1) at the T1 stage. (**B**) Venn diagram showing the overlapping up-regulated DEGs of GA_3_ vs. CK (G2 vs. C2) and PAC vs. CK (P2 vs. C2), and down-regulated DEGs of GA_3_ vs. CK (G2 vs. C2) and PAC vs. CK (P2 vs. C2) at the T2 stage. (**C**) Venn diagram showing the overlapping up-regulated DEGs of GA_3_ vs. CK (G3 vs. C3) and PAC vs. CK (P3 vs. C3), and down-regulated DEGs of GA_3_ vs. CK (G3 vs. C3) and PAC vs. CK (P3 vs. C3) at the T3 stage. (**D**) Venn diagram showing the overlapping up-regulated DEGs of GA_3_ vs. CK (G4 vs. C4) and PAC vs. CK (P4 vs. C4), and down-regulated DEGs of GA_3_ vs. CK (G4 vs. C4) and PAC vs. CK (P4 vs. C4) at the T4 stage.

The FPKM method was employed to analyze the DEGs across the different stages under three treatments. Through pairwise comparisons, we identified a total of 2067 (1596 up- and 471 down-regulated) for G1 vs. G0, 2305 (1805 up- and 500 down-regulated) for C1 vs. C0, 2235 (1799 up- and 436 down-regulated) for P1 vs. P0, 1338 (496 up- and 842 down-regulated) for G1 vs. C1, 1449 (664 up- and 785 down-regulated) for P1 vs. C1, 2014 (1672 up- and 342 down-regulated) for G2 vs. C2, 4727 (1991 up- and 2736 down-regulated) for P2 vs. C2, 6226 (4073 up- and 2153 down-regulated) for G3 vs. C3, 9276 (2680 up- and 6596 down-regulated) for P3 vs. C3, 4014 (3033 up- and 981 down-regulated) for G4 vs. C4, and 7472 (1889 up- and 5583 down-regulated) for P4 vs. C4. In addition, a Venn diagram was used to analyze the overlap among the different comparison groups. Co-expressed DEGs were not identified among the four comparison groups of GA_3_ vs. CK and PAC vs. CK at the same time point (Figure 7). However, 583, 296, 998, and 486 DEGs were co-expressed among two comparison groups: GA_3_-T1 vs. CK-T1 and PAC-T1 vs. CK-T1, GA_3_-T2 vs. CK-T2 and PAC-T2 vs. CK-T2, GA_3_-T3 vs. CK-T3 and PAC-T3 vs. CK-T3, and GA_3_-T4 vs. CK-T4 and PAC-T4 vs. CK-T4 (Figure 8). These results suggested that the co-expressed DEGs might play an essential role in the GA_3_-induced germination of seeds.

### 3.9. DEGs Related to Starch and Sucrose Metabolism in Seed Germination

**Figure 9:**
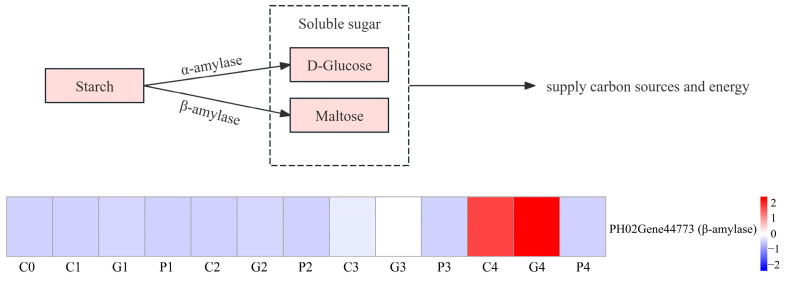
Analysis of DEGs involved in the metabolism of starch. G, C, and P represents GA_3_, CK, and PAC treatments, while 0–4 represent T0–T4 stages, respectively. Expression levels ranging from blue to red indicate low to high expression of gene.

Once seed germination is activated, stored reserves, especially starch, are mobilized to provide nutrients. This process mainly depends on the amylase, including α-amylase and β-amylase. However, the physiological results suggested that starch decomposition was predominantly associated with β-amylase. Four DEGs annotated as β-amylase were identified. Among them, PH02Gene44773 were gradually upregulated during the T0 to T4 stages, with the following order of expression: GA_3_ > CK > PAC (Figure 9).

### 3.10. DEGs Involved in the Antioxidant System

**Figure 10:**
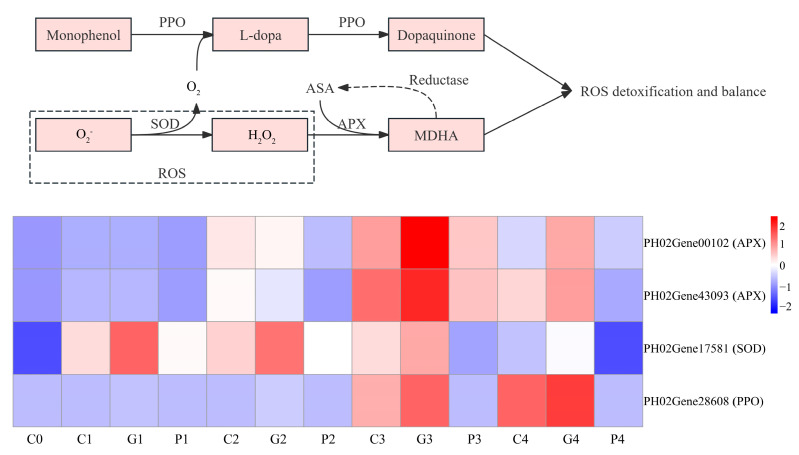
Analysis of DEGs involved in the metabolism of antioxidant enzymes. G, C, and P represents GA_3_, CK, and PAC treatments, while 0–4 represent T0–T4 stages, respectively. Expression levels ranging from blue to red indicate low to high expression of genes.

Antioxidants play a vital role in seed germination. Therefore, we analyzed the expression of genes involved in the antioxidant enzymes. A total of four DEG-related genes were identified from transcriptome data, including one SOD, one PPO, and two APX. The APX family (PH2Gene00102 and PH02Gene43093) was found to be upregulated following different soaking treatments, with their expression levels under GA_3_ and CK treatments significantly exceeding those under the PAC treatment. At the T1 and T2 stages, the expression of these APX genes under the CK treatments was higher than that under GA_3_ treatment; nevertheless, an opposite trend was observed at the T3 and T4 stages. In addition, a SOD family gene (PH02Gene17581) was upregulated following different soaking treatments, with expression level following the order of GA_3_ > CK > PAC. The PPO family gene (PH02Gene28608) was upregulated after GA_3_ treatment, while its expression exhibited irregular trend under CK and PAC treatments (Figure 10).

### 3.11. DEGs Related to Hormone in Seed Germination

**Figure 11:**
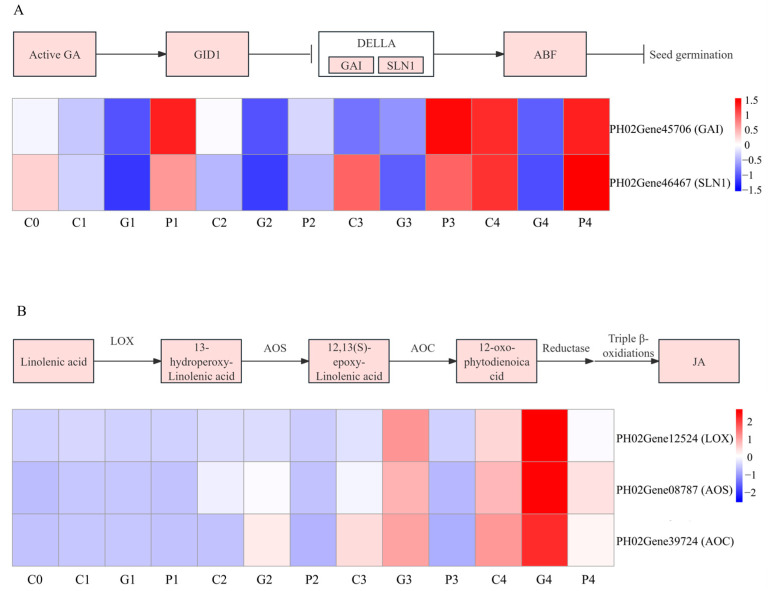
Analysis of DEGs involved in the metabolism of GA signal transduction (**A**) and JA synthesis (**B**). G, C, and P represents GA_3_, CK, and PAC treatments, while 0–4 represent T0–T4 stages, respectively. Expression levels ranging from blue to red indicate low to high expression of gene.

The physiological results indicated that GA_3_ and JA were closely correlated with the seed germination of *P. edulis* by exogenous GA_3_ treatment. The KEGG annotation results also demonstrated that DEGs involved in plant hormone signal transduction were significantly correlated with GA_3_-induced seed germination. To identify the DEGs significantly associated with the response to GA_3_ treatment, DEGs related to the signal transduction pathways of GA and JA were analyzed in this study. In the GA biosynthesis pathway, GID1 and DELLA play major roles. The DELLA family (PH02Gene45706 and PH02Gene46467) was downregulated compared with the T0 stage, and their expression in the GA_3_ treatment was lower than in other treatments. However, the GID1 family was not found (Figure 11A). In the JA biosynthesis pathway, the expression of lipoxygenase (LOX), allene oxide synthase (AOS), and allene oxide cyclase (AOC) exert a vital role in regulating the JA level. In total, five LOX, five AOS, and one AOC genes were identified. Among them, PH02Gene12524 (LOX), PH02Gene08787 (AOS), and PH02Gene39724 (AOC) were upregulated at the T2 to T4 stages, with the order of expression being GA_3_ > CK > PAC (Figure 11B).

## 4. Discussion

### 4.1. Exogenous GA_3_ Promotes Seed Germination of P. edulis by Enhancing Metabolism of Storage Reserves

Given the positive regulatory role of GA in seed germination, the application of exogenous GA_3_ has been shown to accelerate the germination of most seeds [46,47]. In our study, exogenous GA_3_ significantly improved the germination rate and germination index of *P. edulis* seeds, conforming to the findings reported by Li et al. [33]. Soluble sugar and amino acid serve as energy sources during the seed germination process [3,48]. Exogenous GA_3_ can induce the activity of amylase and protease in seeds of grasses including rice and wheat, which therefore accelerates the utilization of starch and protein, in turn promoting seed germination [11,49]. In our experiments, the content of soluble protein decreased at the T1–T2 stage compared to the T0 stage, and was lower in GA_3_-treated seeds compared with the CK treatment. Meanwhile, the protease activity increased during germination in GA_3_-treated seeds. These results indicated that exogenous GA_3_ promoted the hydrolysis of soluble protein by inducing protease activity in the early stage of *P. edulis* seed germination.

Starch serves as a stable energy reserve that can continuously release soluble sugar, which responds quickly to the immediate energy demands of seed germination. Soluble sugar also plays an essential role in osmotic regulation and signal transduction, thereby ensuring the coordinated functioning of energy supply, water absorption, and metabolic regulation during the seed germination process. This coordination facilitates smooth germination and the growth of healthy seedlings. Previous studies indicated that α-amylase is the main enzyme involved in starch hydrolysis, while β-amylase also contributes to starch mobilization. Exogenous GA_3_ accelerates this process by enhancing the activities of both enzymes [49,50,51,52]. In *Dendrocalumus brandisii*, α-amylase played the key role in starch degradation as compared to β-amylase during seed germination [53]. Differently, GA_3_ treatment significantly increased β-amylase activity, while no significant increase in α-amylase activity was observed in our study. Meanwhile, the change in β-amylase activity was accompanied by a significant decrease in starch content under GA_3_ treatment compared with other treatments during the seed germination of *P. edulis*. Similar findings were reported in *Suaeda glauca* seeds [54]. In addition, we found that a β-amylase gene was significantly upregulated. These results suggested that the enhanced β-amylase activity contributed to more efficient starch metabolism and improved energy supply during germination of *P. edulis* seeds under exogenous GA_3_ treatment.

### 4.2. Exogenous GA_3_ Accelerates Seed Germination of P. edulis by Enhancing Antioxidant Capacity

During imbibition and germination, seeds produce excessive reactive oxygen species (ROS) that can damage cellular structures [55]. However, antioxidant enzymes including SOD, PPO, and APX can scavenge free radicals and ROS, maintain the balance between ROS generation and elimination, prevent lipid-membrane peroxidation, and mitigate or reduce plant injury, thereby ensuring that physiological processes proceed smoothly throughout seed germination [55,56]. MDA, an indicator of oxidative stress, is considered as one of the key factors inhibiting seed germination, as it exacerbates damage to cellular membranes [57,58]. In *P. edulis* seeds, exogenous GA_3_ significantly reduced MDA level throughout germination, whereas PAC treatment results in a marked increase. GA_3_ is generally known to enhance the antioxidant enzyme system, thereby improving seed germination performance [59]. Several studies have confirmed the positive effects of GA_3_ [60,61]. Our results suggested that GA_3_-treated seeds exhibited elevated activities of SOD, PPO, and APX across most stages of germination. Conversely, PAC suppressed these enzyme activities, indicating that compromised ROS detoxification contributed to the failure of PAC-treated seeds to complete germination. Transcriptomic profiling identified five genes whose expression patterns mirrored the enzymatic dynamics of SOD, PPO, and APX. These data suggested that GA_3_ not only enhanced antioxidant activity at the biochemical level but also transcriptionally up-regulated the corresponding genes, thereby reinforcing the ROS-scavenging machinery during *P. edulis* germination. Analogous GA_3_-mediated antioxidant priming has been reported in other species [62].

### 4.3. Exogenous GA_3_ Regulates Seed Germination by Endogenous Hormonal Balance During Germination

Seed germination is controlled by a sophisticated hormonal network in which endogenous phytohormones interact synergistically or antagonistically [63,64]. Among these hormones, GA and ABA are the principal antagonists: GA_3_ accelerates germination, whereas ABA inhibits it [65,66]. In this study, the GA_3_ content in dry seeds was significantly lower and increased rapidly after soaking, followed by a decrease and a subsequent increase during the germination of *P. edulis* seeds. ABA content significantly increased at the T1 stage and significantly decreased at the T2–T4 stages compared with dry seeds, while no significant difference was observed between GA_3_ and CK treatments at the T2 and T3 stages. These data suggested that exogenous GA_3_ promoted early germination of *P. edulis* seeds by inducing endogenous GA_3_ rather than by suppressing ABA. This finding diverges from the results reported by Luo et al. [67]. Therefore, GA and ABA play extremely vital roles in the process of seed germination, and they are not the sole regulatory factors determining the process. Except GA and ABA, other hormones including JA and IAA also modulate seed germination through their unique signaling pathways [68]. Exogenous JA has been shown to stimulate the seed germination in *Pyrus communis* and *Triticum aestivum* [69,70]. In our experiment, the JA content in *P. edulis* seeds was significantly increased after soaking and continued to rise from T1 to T4 stages under GA_3_ treatment, surpassing both CK and PAC treatments. IAA has traditionally been considered as a germination inhibitor [64]. Exogenous IAA can delay the seed germination of wheat and Glycine max [71]. Our results demonstrated that the IAA content in *P. edulis* seeds significantly accumulated after soaking, except at the T4 stage under GA_3_ treatment, while it remained lower in GA_3_-treated seeds at the T1, T2, and T4 stages. This finding is inconsistent with the previous reports [71]. Collectively, these results suggested that exogenous GA_3_ promoted the seed germination of *P. edulis* by elevating endogenous GA_3_ and JA levels.

To elucidate the molecular basis of these hormonal changes, transcriptome profiling was conducted. *GID1*, as a receptor for GA_3_, forms a complex with GA_3_, leading to the degradation of the growth inhibitor DELLA protein in the GA_3_ signaling pathway [72]. Li et al. reported that neither *GID1-* or *DELLA*-related genes are transcribed in *P. edulis* seeds during the germination [30]. Our RNA-seq data corroborate their finding that no *GID1* homologs are significantly expressed. In contrast, we detected a clear decline in DELLA family (PH02Gene45706 and PH02Gene46467) transcripts compared with the T0 stage, and this reduction was most pronounced under GA_3_ treatment. Therefore, it could be speculated that exogenous GA_3_ promoted the seed germination of *P. edulis* by degrading DELLA family genes rather than depending on *GID* abundance. The same result was also observed in rice [73]. JA biosynthesis is regulated by the sequential actions of LOX, AOS, and AOC [74]. Some related genes were identified by DEGs analysis. Transcriptomic analysis revealed three corresponding genes including PH02Gene12524 (LOX), PH02Gene08787 (AOS), and PH02Gene39724 (AOC), whose expression increased from T2 to T4, following the order GA_3_ > CK > PAC, mirroring the JA content profile. In summary, exogenous GA_3_ accelerates the seed germination of *P. edulis* by influencing the related genes of GA_3_ signaling pathway and JA biosynthesis.

## 5. Conclusions

**Figure 12:**
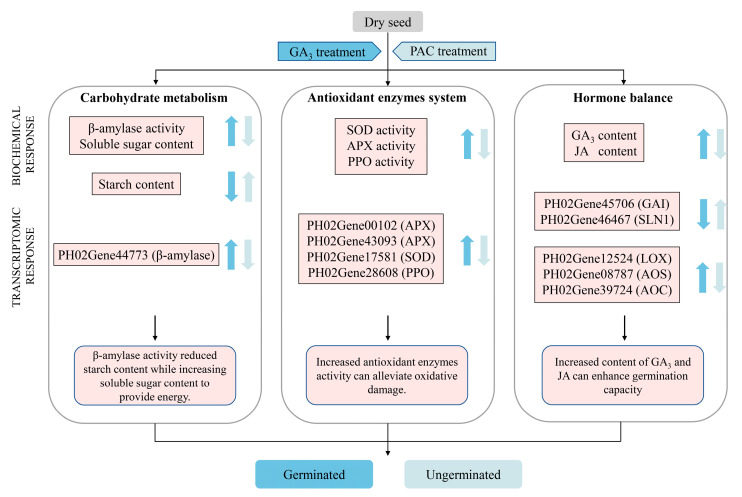
Regulation model of exogenous GA_3_ and PAC treatment on germination of *P. edulis* seeds. Blue color represents GA_3_ treatment, while light blue color represents PAC treatment. The arrows pointing upward refer to up-regulated responses, while the arrows pointing downward refer to down-regulated responses.

To conclude, our study compared the physiological and transcriptional differences in *P. edulis* seeds subjected to CK, GA_3_, and PAC treatments. A regulatory model related to exogenous GA_3_ and PAC treatments on germination was proposed according to the PCA of physiological indicators and transcriptome analysis (Figure 12). The results demonstrate that the application of exogenous GA_3_ effectively activates the activities of β-amylase, thereby significantly reducing the concentrations of starch, while increasing the content of soluble sugar, different from other reported bamboo. Moreover, the exogenous GA_3_ treatment significantly enhances the activities of SOD, PPO, and APX during seed germination, further contributing to the clearance of ROS during the seed germination of *P. edulis*. Interestingly, exogenous GA_3_ promotes the seed germination of *P. edulis* by affecting GA_3_ and JA level rather than ABA content. Through RNA-seq analysis, the related DEGs of GA signaling pathway and JA biosynthesis are found. Notably, two DELLA homologs were identified; however, no *GID1* homolog could be found. This indicates that DELLA acts as an immediate GA sensor during the seed germination of *P. edulis* by responding to exogenous GA_3_. The results of our study will help us further understand the physiological and molecular mechanism underlying GA_3_-induced germination in *P. edulis* seeds.

## Figures and Tables

**Table 1:** Germination parameters of *P. edulis* seeds in each treatment.

Treatment	Germination Percentage (GP, %)	Germination Energy (GE, %)	Germination Index (GI)	Mean Germination Time (MGT, d)
CK	41.0 ± 3.0 b	36.6 ± 0.2 a	1.9 ± 0.1 b	15.6 ± 3.7 a
GA_3_	52.0 ± 6.0 a	38.9 ± 4.5 a	2.5 ± 0.2 a	14.8 ± 3.5 a
PAC	0.0 c	0.0 b	0.0 c	0.0 b

The same letter within the same column represents a non-significant difference at the *p* > 0.05 level, and different letters indicate a significant difference at the *p* < 0.05 level. Values are means value of three biological replicates (*n* = 3) ± standard error (SE).

## Data Availability

All data generated in this research is included within the article. The raw data of RNA-seq was deposited in NCBI SRA under accession number PRJNA1307053.

## Appendix A

### Appendix A.1

**Table A1:** Eigen value, variability percentage, and PCA loading of 14 parameters.

Parameters	PC1	PC2
ABA	−0.21797	0.4092
JA	0.34125	−8.03 × 10^−5^
IAA	0.1037	−0.51327
GA	0.31237	0.22282
soluble protein	−0.12324	−0.32462
soluble sugar	0.3307	0.12852
starch	−0.30555	−0.12266
α-amylase	−0.17591	0.38624
β-amylase	0.3388	0.08431
protease	0.21537	0.40345
APX	0.32568	−0.13951
PPO	0.30915	−0.20541
SOD	0.34063	0.04198
MDA	0.0025	−0.00596
Eigen value	8.4804	3.37035
Variability %	60.6	24.1

**Table A2:** RNA-seq data quality statistical analysis.

Sample	Raw Reads	Clean Reads	Clean Data (bp)	Q20 (%)	Q30 (%)	Total Mapped Reads	Multiple Mapped Reads	Uniquely Mapped Reads
T0-1	52,850,320	50,536,670	7,631,037,170	97.54	93.78	46,064,556 (91.15%)	2,795,073 (6.07%)	43,269,483 (93.93%)
T0-2	48,653,908	46,540,196	7,027,569,596	97.41	93.49	42,343,218 (90.98%)	2,524,403 (5.96%)	39,818,815 (94.04%)
T0-3	48,408,030	46,183,684	6,973,736,284	97.47	93.65	42,042,215 (91.03%)	2,490,463 (5.92%)	39,551,752 (94.08%)
G1-1	42,249,322	40,316,952	6,087,859,752	97.50	93.89	36,460,133 (90.43%)	2,440,356 (6.69%)	34,019,777 (93.31%)
G1-2	51,810,176	49,531,224	7,479,214,824	97.30	93.35	44,541,094 (89.93%)	3,173,131 (7.12%)	41,367,963 (92.88%)
G1-3	49,680,734	47,535,422	7,177,848,722	97.49	93.80	42,816,098 (90.07%)	2,906,413 (6.79%)	39,909,685 (93.21%)
C1-1	41,683,570	39,821,840	6,013,097,840	97.44	93.67	36,371,209 (91.33%)	2,156,916 (5.93%)	34,214,293 (94.07%)
C1-2	45,461,818	43,406,602	6,554,396,902	97.51	93.76	39,703,789 (91.47%)	2,294,386 (5.78%)	37,409,403 (94.22%)
C1-3	50,133,194	47,931,194	7,237,610,294	97.44	93.57	43,801,747 (91.38%)	2,536,452 (5.79%)	41,265,295 (94.21%)
P1-1	45,764,410	43,754,092	6,606,867,892	97.45	93.74	39,535,032 (90.36%)	2,387,472 (6.04%)	37,147,560 (93.96%)
P1-2	52,310,254	49,955,160	7,543,229,160	97.49	93.83	45,163,069 (90.41%)	2,791,129 (6.18%)	42,371,940 (93.82%)
P1-3	43,863,914	41,927,410	6,331,038,910	97.56	93.99	37,890,248 (90.37%)	2,316,271 (6.11%)	35,573,977 (93.89%)
G2-1	43,282,354	41,091,190	6,204,769,690	97.79	94.10	38,647,132 (94.05%)	1,920,983 (4.97%)	36,726,149 (95.03%)
G2-2	44,857,842	42,603,580	6,433,140,580	97.71	93.94	40,050,531 (94.01%)	1,970,855 (4.92%)	38,079,676 (95.08%)
G2-3	52,885,574	50,197,296	7,579,791,696	97.67	93.76	47,217,057 (94.06%)	2,301,076 (4.87%)	44,915,981 (95.13%)
C2-1	52,206,732	49,578,412	7,486,340,212	97.86	94.27	46,635,778 (94.06%)	2,357,648 (5.06%)	44,278,130 (94.94%)
C2-2	50,077,694	47,553,058	7,180,511,758	97.67	93.81	44,567,062 (93.72%)	2,328,859 (5.23%)	42,238,203 (94.77%)
C2-3	43,962,458	41,740,470	6,302,810,970	97.87	94.35	39,151,798 (93.80%)	2,021,445 (5.16%)	37,130,353 (94.84%)
P2-1	43,728,180	41,576,904	6,278,112,504	97.71	93.97	39,021,453 (93.85%)	1,991,669 (5.10%)	37,029,784 (94.90%)
P2-2	52,331,552	49,740,852	7,510,868,652	97.73	93.96	46,679,366 (93.85%)	2,428,439 (5.20%)	44,250,927 (94.80%)
P2-3	46,137,400	43,821,376	6,617,027,776	97.74	94.03	41,070,278 (93.72%)	2,114,680 (5.15%)	38,955,598 (94.85%)
G3-1	51,017,512	48,426,990	7,312,475,490	97.81	94.13	45,666,601 (94.30%)	2,429,011 (5.32%)	43,237,590 (94.68%)
G3-2	52,810,726	50,191,392	7,578,900,192	97.83	94.19	47,277,070 (94.19%)	2,574,813 (5.45%)	44,702,257 (94.55%)
G3-3	48,324,224	45,881,938	6,928,172,638	97.94	94.50	43,267,940 (94.30%)	2,305,947 (5.33%)	40,961,993 (94.67%)
C3-1	46,349,158	44,046,918	6,651,084,618	97.80	94.13	41,497,948 (94.21%)	1,978,000 (4.77%)	39,519,948 (95.23%)
C3-2	55,469,422	52,704,006	7,958,304,906	97.66	93.73	49,640,645 (94.19%)	2,351,790 (4.74%)	47,288,855 (95.26%)
C3-3	51,160,342	48,577,058	7,335,135,758	97.76	94.05	45,711,769 (94.10%)	2,196,002 (4.80%)	43,515,767 (95.20%)
P3-1	49,846,126	46,988,048	7,095,195,248	97.67	93.90	44,228,852 (94.13%)	2,685,693 (6.07%)	41,543,159 (93.93%)
P3-2	48,311,390	45,666,704	6,895,672,304	97.88	94.31	43,182,964 (94.56%)	2,509,117 (5.81%)	40,673,847 (94.19%)
P3-3	49,833,424	47,062,108	7,106,378,308	97.74	94.05	44,447,195 (94.44%)	2,607,219 (5.87%)	41,839,976 (94.13%)
G4-1	42,391,122	40,001,676	6,040,253,076	97.94	94.27	38,291,331 (95.72%)	1,658,599 (4.33%)	36,632,732 (95.67%)
G4-2	49,381,724	46,561,012	7,030,712,812	98.15	94.89	44,629,577 (95.85%)	1,926,068 (4.32%)	41,839,976 (94.13%)
G4-3	50,275,080	47,442,424	7,163,806,024	98.12	94.68	45,551,568 (96.01%)	1,993,706 (4.38%)	43,557,862 (95.62%)
C4-1	47,916,340	45,559,088	6,879,422,288	97.95	94.35	43,230,807 (94.89%)	1,966,982 (4.55%)	41,263,825 (95.45%)
C4-2	47,201,546	44,828,862	6,769,158,162	97.75	93.99	42,284,995 (94.33%)	2,051,412 (4.85%)	40,233,583 (95.15%)
C4-3	47,873,136	45,480,484	6,867,553,084	97.92	94.39	42,970,739 (94.48%)	2,083,979 (4.85%)	40,886,760 (95.15%)
P4-1	42,168,746	40,052,536	6,047,932,936	97.77	94.22	37,507,410 (93.65%)	1,993,925 (5.32%)	35,513,485 (94.68%)
P4-2	51,069,950	48,628,690	7,342,932,190	97.73	93.96	45,593,820 (93.76%)	2,399,789 (5.26%)	43,194,031 (94.74%)
P4-3	47,269,230	44,969,746	6,790,431,646	97.66	93.85	41,932,518 (93.25%)	2,346,052 (5.59%)	39,586,466 (94.41%)
